# A Two-Mode Underwater Smart Sensor Object for Precision Aquaculture Based on AIoT Technology

**DOI:** 10.3390/s22197603

**Published:** 2022-10-07

**Authors:** Chin-Chun Chang, Naomi A. Ubina, Shyi-Chyi Cheng, Hsun-Yu Lan, Kuan-Chu Chen, Chin-Chao Huang

**Affiliations:** 1 Department of Computer Science and Engineering, National Taiwan Ocean University, Keelung City 202, Taiwan; 2 College of Computing Studies, Information and Communications Technology, Isabela State University, PM9Q+C9F, Echague 3309, Philippines; 3 Department of Aquaculture, National Taiwan Ocean University, Keelung City 202, Taiwan

**Keywords:** sonar images, stereo RGB images, Mask R-CNN, gaussian mixture models, convolutional neural networks, semantic segmentation networks, object detection CNN

## Abstract

Monitoring the status of culture fish is an essential task for precision aquaculture using a smart underwater imaging device as a non-intrusive way of sensing to monitor freely swimming fish even in turbid or low-ambient-light waters. This paper developed a two-mode underwater surveillance camera system consisting of a sonar imaging device and a stereo camera. The sonar imaging device has two cloud-based Artificial Intelligence (AI) functions that estimate the quantity and the distribution of the length and weight of fish in a crowded fish school. Because sonar images can be noisy and fish instances of an overcrowded fish school are often overlapped, machine learning technologies, such as Mask R-CNN, Gaussian mixture models, convolutional neural networks, and semantic segmentation networks were employed to address the difficulty in the analysis of fish in sonar images. Furthermore, the sonar and stereo RGB images were aligned in the 3D space, offering an additional AI function for fish annotation based on RGB images. The proposed two-mode surveillance camera was tested to collect data from aquaculture tanks and off-shore net cages using a cloud-based AIoT system. The accuracy of the proposed AI functions based on human-annotated fish metric data sets were tested to verify the feasibility and suitability of the smart camera for the estimation of remote underwater fish metrics.

## 1. Introduction

Aquaculture, with the aim of the farmed production of fish or shellfish, has been one of the great contributors to supplying fish or seafood products for human consumption. In 1974, aquaculture contributed 7% of the fish supply and reached 50% in 2020 [1]. With this vast increase in production and to cope with the supply–demand due to the increasing population, many aquaculture farms are expanding rapidly. However, these expansions require effective fish farm management, which is much needed to solve relevant aquaculture issues, including environmental degradation, disease and parasite outbreaks, labor shortage, and productivity maximization by efficiently managing its resources. These challenges can be addressed by integrating fish farm monitoring-based technology with Artificial Intelligence-based Internet of Things (AIoT). At the same time, machine learning and big-data analytics make it possible to collect, process, and analyze large volumes of heterogeneous datasets. When combined, these powerful technologies craft a precision aquaculture framework that uses sensors, cloud, and analytics to enable real-time, evidence-based decision-making to optimize operations [2].

Precision aquaculture requires adopting technologies such as information-based management with big data and models to guide the production process [3] to fully understand the environmental and fish conditions in the cage. Its goal is to enable farmers to make intelligent decisions by providing objective information to improve their capability to monitor and control factors that involve fish production; thus, farming decisions are adjusted to improve fish health and maximize farm production. Large and modern aquaculture farms must incorporate technological innovation to automate their processes, minimize workforce requirements, and maximize their fish feeding process. It enables farmers to integrate technology and data-driven decisions making, enabling efficient aquaculture farm management and remote monitoring, especially for farms situated in the open sea. The use of machine learning and computer vision in Artificial Intelligence (AI), together with sensors and Internet of Things (IoT) technologies, have been widely used to monitor fish feeding behavior, disease, and growth as a non-invasive method, thereby enabling objective observation of the fish farm. Such a mechanism also allows data collection and real-time image acquisition using reliable wireless communication channels [4] without relying so much on human intervention [5].

Various sensors such as temperature, position, humidity, flow, and photo optic or camera sensors have changed how the world accesses data from remote locations. These devices have bridged the gap in collecting data from the physical environment and transmitting wirelessly to a platform with a network of remote servers for storage, management, and data processing [6]. Data collection from physical environments can be carried out by other means such as underwater vehicles [7]. The advancement of cloud computing and IoT has brought tremendous innovation and improvement to aquaculture farming. Cloud computing services enable the collection and storage of big data for processing using AI methodologies capable of predictive analysis to provide informed decision-making mechanisms for precise aquaculture. It enables a brand-new farming approach [8] that eases the burden of the farming industry in terms of monitoring.

For aquaculture farms, it is vital to monitor the fish growth and population as an essential parameter to approximate fish food and assess the overall wellness of the fish species. To achieve the goal of smart aquaculture, fish counting and body length estimation using underwater images are essential to estimate the fish growth curve [9,10]. Cameras as sensors can now be used to capture underwater fish images in an off-shore cage in a non-intrusive manner that reduces the manual handling of the fish, thus reducing direct contact that can cause stress, injury, and growth disturbance to the fish species in the cage. In addition, sonar and stereo cameras for data collection and computer vision can estimate the fish’s biological information. Sonar and RGB cameras, such as stereo systems, are just one of the most widely used and studied systems for underwater environment monitoring.

**Figure 1:**
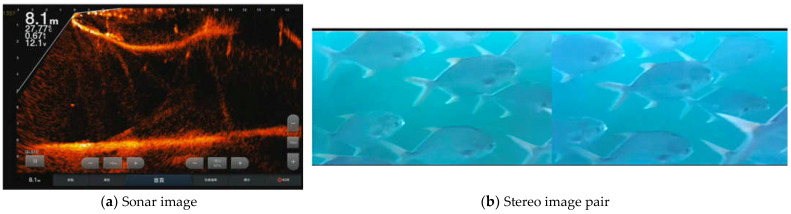
Data captured from the sensor devices is transmitted to the cloud for storage and big data analytics.

In the underwater environment where the lighting condition is poor or low, RGB cameras are limited. In contrast, sonar cameras are more robust concerning the issue of light attenuation and water turbidity that severely affects optical sensors. In terms of area to cover in capturing the underwater environment, sonar cameras have more scope and a higher range than stereo cameras, as shown in Figure 1. In addition, a sonar camera provides a depth reference value for 3D images, further improving the length or size estimation accuracy. Various studies also dealt with using sonar systems and their applicability to fish length estimation [11,12,13]. A 3D sonar camera allows direct representation of a scene’s 3D information, drastically reducing or no longer requiring the 3D reconstruction process from 2D views, making it more viable for real-time data processing [14]. However, the cost of a high-resolution 3D sonar camera is expensive. To meet the cost concerns of sensors for aquaculture management, in this study, we proposed a fusion of using a low-cost sonar imaging device and a stereo camera system for aquaculture fish monitoring.

**Figure 2:**
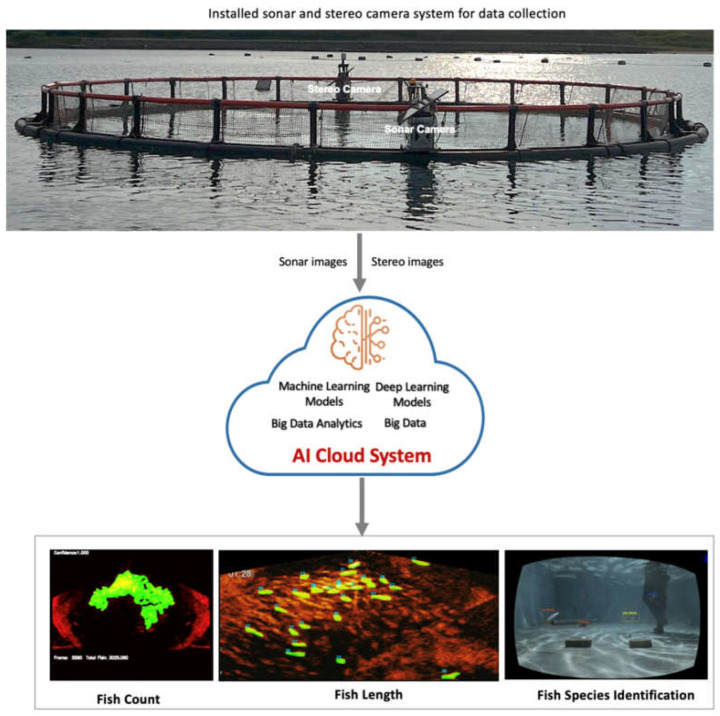
The framework of our proposed AIoT technology for smart Underwater surveillance for precision aquaculture.

Figure 2 shows the framework of our AIoT system where the two camera sensors (sonar and stereo camera) were deployed and installed in the aquaculture farm to collect images/videos from the site. In addition, these sensors were equipped with wireless transmission capabilities to send the collected data to the AI cloud services, where each of the trained deep learning and machine learning models performed the necessary AI function for a specific application.

Our proposed sensor fusion comprises four steps: data collection from the aquaculture sites using our sonar and stereo camera system sensors, 3D-point cloud estimation, overlapping detection, and object detection to integrate AI functions; the details of these are discussed in the subsequent section. In this work, we used the sonar camera system as the primary sensor device for collecting depth information from the target fish objects to perform fish metric estimation, specifically for fish length and fish count. It uses its beam to collect fish information by sending multiple sound waves to the scene. Thus, it can capture the real environment’s depth information. Sonars use depth information to form an image object much different from an optical image.

Although sonar devices, as mentioned earlier, provide bigger coverage, they do not have texture and color information since they just provide depth information. Due to refraction, the shape of the captured fish sonar images is affected. Thus, they can only map macro-features due to their limited resolution [15]. The stereo camera system addresses this concern or limitations of the sonar camera system. These two devices can work together to provide a clearer picture of the underwater fish object in the aquaculture cages or ponds. Since sonar images lack color information, we used the RGB images captured from the low-cost sonar camera to provide additional functions for fish-type annotation.

One of the challenges of sensor fusion is to detect the common area of each sensor or their corresponding images, and considering the environment is underwater, more problems arise. Additionally, the target objects in the sonar and stereo camera systems have different positions, so a mechanism should be devised to project the same target image into the same plane. Incorporating transformation in their corresponding rotation and translation vectors will map the features from sonar to optical coordinate system using the extrinsic sensor calibration method. Since each sensor’s range is different, the target object and its corresponding shape should be recognized by both [14]. In this work, we proposed a method to determine the sensor’s overlapping areas using their corresponding 3D point cloud information so that the sonar images are projected to the stereo images. We used four markers (four pixels) or points with their corresponding 3D point sets. Both the sonar and stereo camera systems should be able to detect or distinguish these markers. We had two phases to integrate plane projection conversion. First, the learning phase obtained the transformation matrix in rotation and translation based on the marker’s information. Second, each pixel in the sonar image was transformed into its corresponding pixel in the stereo image (left image). Then, the result of the transformation was projected into the 2D space to identify the pixel correspondence for both camera systems. In the testing phase, for each pixel in the frame of the sonar camera, the transformation matrices were then used to locate the corresponding pixel in the synchronized frame of the optical camera. Thus, the common area covered by the sonar and RGB cameras was detected.

The contributions of our paper are the following:

- We proposed an AIoT system that provides sonar and stereo camera fusion that supports automatic data collection from aquaculture farms and performs artificial intelligence functions such as fish type detection, fish count, and fish length estimation. To our knowledge, combining a low-cost sonar and stereo camera system tested in various aquaculture environments with different AI monitoring functions is a novel work.
- We designed a methodology to perform sonar and stereo camera system fusion. However, deploying IoT can be expensive, and to limit the cost of its implementation, we employed low-cost sensors that do not entail high additional expenses for aquaculture farmers.
- Using a sonar camera system, we developed our mechanism to estimate the fish’s length and weight. Additional plugin AI functions can also be deployed in the cloud to meet the emerging requirements of decision-making for aquaculture management based on the collected big data sets. Agile development realizes the design of learnable digital agents to achieve the goal of precision aquaculture.

The paper is structured as follows: Section 2 provides the related works, and Section 3 contains the materials and methods, which detail our approach to addressing the issue discussed earlier. Section 4 and Section 5 include the experimental results and discussion, respectively. Finally, the last section outlines our conclusions and recommendations for future works.

## 2. Related Works

IoT and AI have gained popularity in the past few years due to their efficiency and promising results in various fields. For example, in aquaculture production, they have been widely used to improve the accuracy and precision of farming operations, facilitate autonomous and continuous monitoring, provide a reliable decision support system, and limit manual labor demand and subjective assessment of fish conditions [16]. In addition, vision cameras such as stereo and sonar systems are popular for computer vision-based problems with the capability of image processing, object detection and classification, and image segmentation.

Imaging sonar systems have been applied to many aquaculture applications, such as fish counting [17,18], estimation of fish length [11], analysis of fish population [19,20,21], fish tracking [22], fish detection [23], monitoring of fish behavior [24], and control of fish feeding [25]. Hightower et al. [12] used multibeam sonar to determine the reliability of length estimates of some fish species. The sonar was positioned approximately 0.5 m above the bottom, and the beam was aimed slightly off the bottom. Additionally, using sonar image analysis and statistical methodologies, a non-invasive procedure using multibeam sonar was used to count and size the fish in a pond. Simulation software was developed to calculate the abundance correction factor, which depends on the transducer beam size based on the pond size [26]. DIDSON acoustic system with an ultra-high-resolution lens was used to evaluate the accuracy and precision of estimating length from images of tethered fish insonified at the side aspect. The device used has a good potential for discriminating sizes among different species [27]. Lagarde et al. [28] integrated an ARIS acoustic camera to perform counts and size estimates for European eels. Count estimates were performed using 58 videos. The acoustic camera was installed in a channel that links a lagoon to the Mediterranean Sea. It was positioned in the narrowest part of the channel and came 53 m wide and 3.5 m deep. A real-time system for scientific fishery biomass estimator was proposed by Sthapit et al. [29] using a compact single beam advanced echosounder. The device, composed of a transducer, processing unit, a keypad, and a display unit, analyzes ping data continuously. In real-time, it calculates various parameters and simultaneously displays the echogram results on the screen.

**Figure 3:**
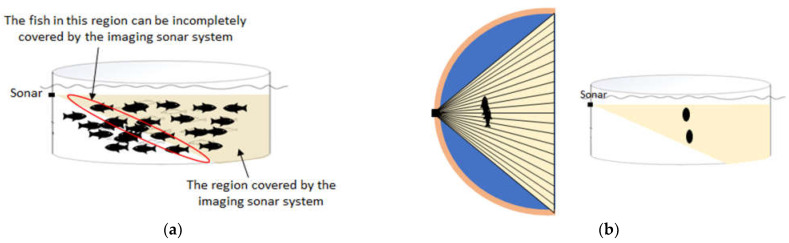
An illustration of the region covered by an imaging sonar system where (**a**) shows that the imaging sonar system can partly cover the fish, and (**b**) shows that the fish at different locations in the direction perpendicular to the sonar beam can be overlapped in the sonar image.

Convolutional Neural Networks, or CNNs, have been applied to process sonar images for many applications, such as detecting objects on the sea floor [30] and counting fish [31]. As illustrated in Figure 3, some characteristics of the fish schools in sonar images are as follows:

- Fish schools swim in the three-dimensional space;
- In the sonar image, fish close to the sonar system can often be incomplete, and fish away from the sonar system become blurrier;
- In sonar images, fish are often overlapped, and the location difference of fish in the direction perpendicular to the sonar beam is indistinguishable [32];
- Annotators are often required to examine successive sonar images to identify fish in sonar images because they find fish by the change of the pattern and strength of echoes.

The stereo camera system has also been extensively used in computer vision. For example, a DeepVision stereo camera system was used by Rosen et al. [33] for continuous data collection of fish color images passing inside the extension of the trawl. Out of 1729 fish captured while trawling, 98% were identified in terms of species. Such a mechanism increases the scope of the information collected specifically on documenting the fine-scale distribution of individual fish and species overlap. The information that can be drawn from this can help interpret acoustic data. The underwater stereo video was also used to determine population counts and spatial and temporal frequencies, incorporating detection and identification [34]. Stereo vision is also integrated for video-based tracking [35], fish volume monitoring [36] or abundance [37], and 3D tracking of free-swimming fish [38].

Stereo camera systems also have been widely used in fish length estimations [39,40,41,42], using disparity information to provide 3D information about an object [43,44,45]. In aquaculture, many are now putting their interest and efforts into integrating stereo cameras for fish length and biomass estimations [40,41,46]. In our previous work, we integrated a low-cost stereo camera system to perform fish metrics estimation. We used a reliable object-based matching using sub-pixel disparity computation with video interpolation CNN and tracked and computed the fish length in each video frame [10].

Through the years, interest in combining various sensors to achieve higher accuracy and efficiency has been widespread. Many studies regarding sensor fusions have been successfully integrated and applied in multiple fields, such as camera-lidar integration for semantic mapping [47], driver aid systems for intelligent vehicles [48,49], target tracking for robotic fish [50], activity detection of sound sources [51] and avian monitoring [52]. An underwater acoustic-optic image matching was proposed by Zhou et al. [53]. Their work combined the advantages of CNN depth features extraction to determine the image visual attribute conversion; the difference between the acousto-optic images was discarded. Their matching technique used current advanced learned descriptions in the generated target image (acoustic) and the original image (optical). The data aggregation method was utilized in displaying the calibrated matching correspondence between the two types of images.

## 3. Materials and Methods

### 3.1. Devices Used and Experimental Environments

**Figure 4:**
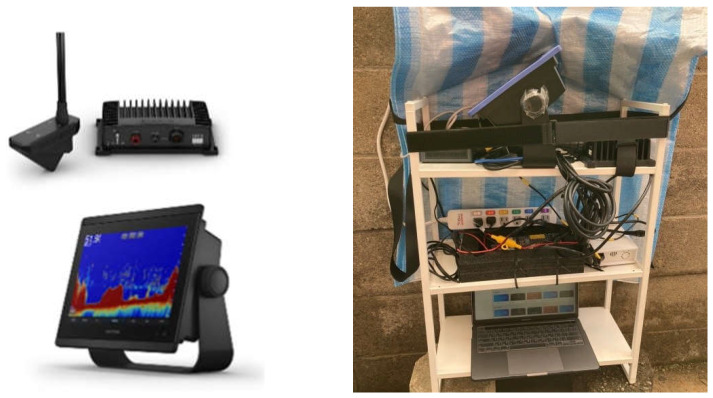
Sonar camera device used for data gathering.

**Figure 5:**
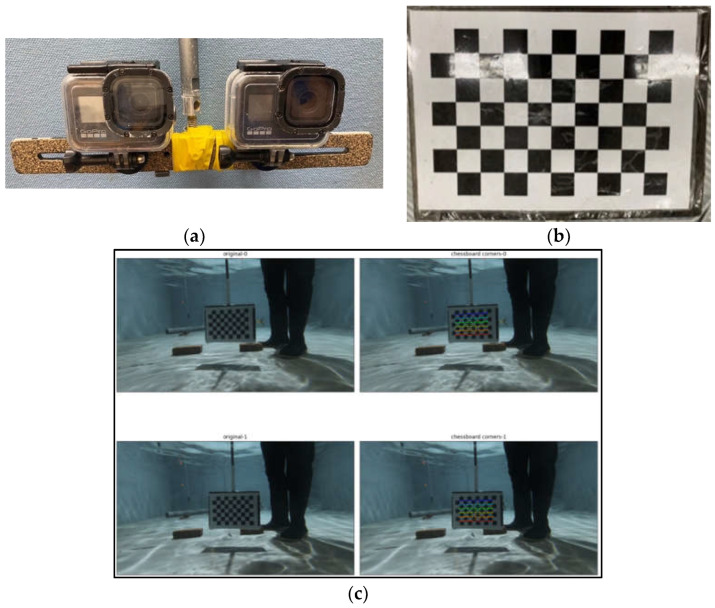
Set-up of the low-cost stereo camera system: (**a**) the stereo camera; (**b**) the correction checkboard for calibrating; (**c**) the stereo camera calibration based on the warping of the check-board map.

Figure 4 shows the sonar equipment we used for the image capture with GARMIN Panoptix LiveScope System (Garmin Ltd., Taiwan), which includes a sonar screen, a processor, and a sonar transducer probe. The sonar system uses an Intel NUC minicomputer (Intel Corporation, Santa Clara, CA, USA) to collect and analyze the sonar images enclosed with the sonar block box. Meanwhile, we used a low-cost camera to set up our stereo camera system using two Go Pro Hero 8 devices (GoPro, San Mateo, CA, USA). The two cameras were mounted in a fixed relative position, as shown in Figure 5a, with a baseline or camera distance of 11 cm. A waterproof case was used to cover the two Go Pro cameras to protect them from water damage since they would be submerged in the water during the data capturing. Next, we calibrated the two stereo cameras using the popular checkboard-based method, as shown in Figure 5b, since the patterns are distinct and easy to detect. The calibration checkboard has an A4 paper size with a 2.5 cm grid size. The first step of the sensor fusion relies on the stereo image rectification process of the left and right images of the low-cost stereo cameras. One potential problem of a low-cost stereo image camera system is that it is incomplete or incorrectly synchronized, causing the object’s pose to be different in the left and right images. As seen in Figure 5c, the checkboard corners serve as the corresponding points of the left and right images.

**Figure 6:**
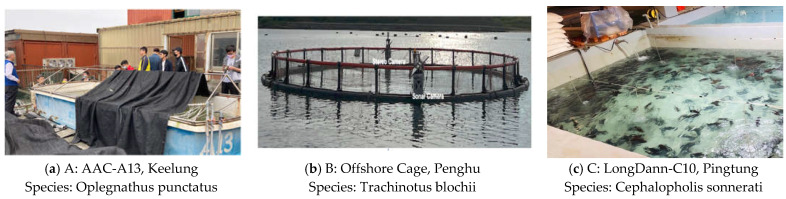
Experimental environments utilized for training various deep learning models for fish length estimation, fish count estimation, and fish type annotation.

The experimental site has three locations representing indoor and outdoor environments and less dense and highly dense fish populations. Figure 6 shows the set-up of the environment and locations with its corresponding fish species. Fish tank A is 4 m in length, 1 m in width, and 0.8 m in depth, and the fish instances are small. Fish tank B is an off-shore cage with 50 m in circumference and 25 m in depth. Lastly, tank C with crowded fish instances is 5.3 m in length, 4 m in width, and 0.8 m in depth.

For simultaneous data capture using the two sensors for calibration, we used a laptop computer as the sonar recording. In contrast, the data captured by the stereo camera were saved in their respective storage devices. The distance between the sonar and stereo camera was 50 cm. The Go Pro camera, which acts as the stereo camera system, used the rg174 signal line to confirm that the target object was captured. In contrast, the sonar was confirmed by the human operators. The computer device that trains the neural networks had an Intel i7-107000k 3.8. GHz CPU, NVIDIA GeForce RTX 3090 GPU, and 48 GB memory.

### 3.2. Sonar and Stereo Camera Fusion

**Figure 7:**
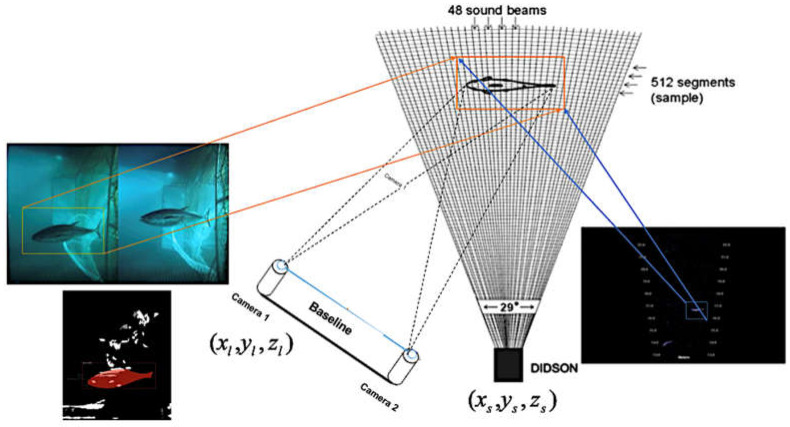
The sensor fusion consists of a stereo camera, and a sonar imaging device captures the fish images from a pond or a net cage. The 3D point clouds of the common object in the left image and the sonar image can be used to calculate the transformation matrix using a 3D affine transformation algorithm [54].

**Figure 8:**
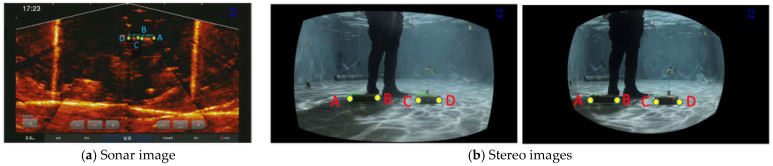
Brick detection and its corresponding four-point marks using YOLOv4 [55] in (**a**) sonar image and (**b**) stereo image pair.

Figure 7 shows the scheme for the sensor fusion that captures underwater images from a fish pond or a net cage. The 3D point clouds of the common object in the left image and the sonar image can be used to calculate the transformation matrix using a 3D affine transformation algorithm [54]. In the training phase, the common object was detected by applying the pre-trained object detection CNN, e.g., YOLOv4 [55] to both the synchronized RGB image and the sonar image. As shown in Figure 8, the bounding boxes of the common object (the brick) were detected from the stereo images and the synchronized sonar image using YOLOv4.

Four markers (A, B, C, D) exist in both sonar and stereo images. Each marked point has an image coordinate of (u, v). To combine sonar and stereoscopic images using camera calibration, we used two bricks as the target object and integrated YOLOv4 to mark or capture the point coordinates provided by the bounding box of the disparity conversion. For the stereo image, the left and the right images were captured and underwent an image rectification process to obtain the correct intrinsic and extrinsic parameters using camera calibration. To find the corresponding points between a stereo pair and plot them into the 3D space, given a point in the left image, its corresponding point in the right image lies on its epipolar line. Using a stereo-image-based disparity matching algorithm in finding the correspondence of the left image to the right image, take a pixel in the left image and search on the epipolar line for that pixel in the right image. The pixel with the minimum cost is selected, and the disparity can now be computed. The point is located on the epipolar line, which would only require a one-dimensional search where cameras need to be aligned along the same axis. To obtain the depth of a stereo image pair, the disparity information is the difference in the image location of the same 3D point projected using two different cameras. The disparity of a pixel $\vec{x}=\left(x,y\right)$ in the left image can be computed by obtaining the difference between

(1) $$d=x-x^{\prime }$$

where $x^{\prime }$ is the x-coordinate of the corresponding pixel in the right image. Once the disparity value of the pixel $\vec{x}$ in the left image has been computed, the depth value from disparity d and its 3D coordinates $X_{O}=(x_{O},y_{O},z_{O}$) can be determined using triangulation:

(2) {zO=f ∗ bd xO=(x−cx) ∗ zOf yO=(y−cy) ∗ zOf 

where f is the focal length of the camera, b is the baseline, defined as the distance between the centers of the left and right cameras, and $\left(c_{x},c_{y}\right)$ is the center of the projected 2D plane.

Similarly, the 3D point P3D (*r*, *θ*, *φ*) of the spherical coordinates where θ is the azimuth direction, and φ is the spread in the elevation direction and can be expressed in Cartesian coordinates as follows:

(3) $$X=\left[\begin{array}{c} x\\ y\\ z \end{array}\right]=\left[\begin{array}{c} r\;cos\;\theta \;\cos\;\emptyset \\ r\;sin\;\theta \;\cos\;\emptyset \\ r\;sin\;\emptyset \; \end{array}\right]$$

The 2D point $\vec{x}=\left(u,v\right)$ projected on the sonar image plane is expressed as follows:

(4) $$\vec{x}=\left[\begin{array}{c} u\\ v \end{array}\right]=\frac{1}{\cos\emptyset }\;\left[\begin{array}{c} x\\ y \end{array}\right]=\left[\begin{array}{c} r\;cos\;\theta \;\\ r\;sin\;\theta \; \end{array}\right]$$

Thus, in the 2D sonar images, the information of the elevation angle and, therefore, the height of information of the target fish objects cannot be identified. For the target object in the underwater environment, critical points refer to the shortest distance points, where the sonar’s acoustic beams reflect on the object. The critical position in the *j*th acoustic beam is expressed as $r_{cp}\left(j\right)$ and $\theta_{cp}\left(j\right)$ using the sonar system’s local coordinate, which can be calculated using:

(5) $$\left[\begin{array}{c} x_{cp}^{Local}\left(j\right)\\ y_{cp}^{Local}\left(j\right)\\ z_{cp}^{Local}\left(j\right) \end{array}\right]=\left[\begin{array}{c} r_{cp}\left(j\right)\;\sqrt{1-sin^{2}\left(t+\frac{s}{2}\right)-sin^{2}\left(\theta_{cp}\left(j\right)\right)\;}\\ r_{cp}\left(j\right)\;sin\left(\theta_{cp}\left(j\right)\right)\\ r_{cp}\left(j\right)\;sin\;\left(t+\frac{s}{2}\right) \end{array}\right]$$

where t and s are the imaging sonar’s tilt angle and spreading angle, respectively. Since the imaging sonar is tilted by an angle t, azimuth angle ${\;\theta }_{cp}\left(j\right)$, it differs from the azimuth angle of the spherical coordinate. Hence, $x_{cp}^{Local}\left(j\right)$ can be calculated using $y_{cp}^{Local}\left(j\right)$ and $z_{cp}^{Local}\left(j\right)$. The critical point’s position in the global coordinates can be expressed using the rotation matrix $R=R_{z}R_{y}R_{x}$ and the position of the imaging sonar $\left(x_{S,W},y_{S,W},z_{S,W}\right)$ in terms of the world coordinate system is represented using:

(6) $$\left[\begin{array}{c} x_{W}\left(j\right)\\ y_{W}\left(j\right)\\ z_{W}\left(j\right) \end{array}\right]=\left[\begin{array}{c} x_{S,W}\\ y_{S,W}\\ z_{S,W} \end{array}\right]+R\left[\begin{array}{c} x_{cp}^{Local}\left(j\right)\\ y_{cp}^{Local}\left(j\right)\\ z_{cp}^{Local}\left(j\right) \end{array}\right]$$

where R represents a 3D rotation transformation matrix to determine the roll angle, pitch angle, and yaw angle of the imaging sonar. The 3D point cloud of the sonar scene can be generated by accumulating the calculated coordinates while scanning [56].

Once the corresponding pixel pairs between the sonar image and the stereo images are detected, the 3D coordinates of the matched points between sonar and stereo images are computed based on the above 3D coordinates computing scheme. Let $X_{O,A}=\left(x_{O,A},y_{O,A},z_{O,A}\right)$ and $X_{S,A}=\left(x_{S,A},y_{S,A},z_{S,A}\right)$ be the 3D coordinates of the common point A generated from the stereo images and the sonar image, respectively. Obviously, $X_{O,A}\neq X_{S,A}$ since they locate point A in different 3D coordinate systems. Let, $X_{W,A}=\left(x_{A},y_{A},z_{A}\right)$ be the 3D coordinates of the point A in the world coordinate system. Then we can apply the following 3D transformation to transform $X_{S,A}$ or $X_{O,A}$ into $X_{W,A}$:

(7) $$X_{W,A}=R_{O\to W}X_{O,A}+T_{O}=R_{S\to W}X_{S,A}+T_{S}$$

where $R_{O\to W}\;$($R_{S\to W}$) is the 3D rotation matrix that aligns the *z*-axis of the optical camera (sonar camera) coordinate system with the *z*-axis of the world coordinate system; $T_{O}\;$($T_{S}$) is the translation vector that locates the center of the optical camera (sonar camera) in the world coordinate system. Equation (7) can be rewritten as

(8) $$X_{O,A}=R_{S\to O}X_{S,A}+T_{S\to O}$$

where $R_{S\to O}=R_{O\to W}^{-1}R_{S\to W}$ and $T_{S\to O}=R_{O\to W}^{-1}\left(T_{S}-T_{O}\right)$. Equation (8) facilitates the computation of the transformation matrices $R_{S\to O}$ and $T_{S\to O}$ by directly matching the set of 3D points, i.e., A, B, C, D points, of the common object in the optical image against that in the sonar image.

**Figure 9:**
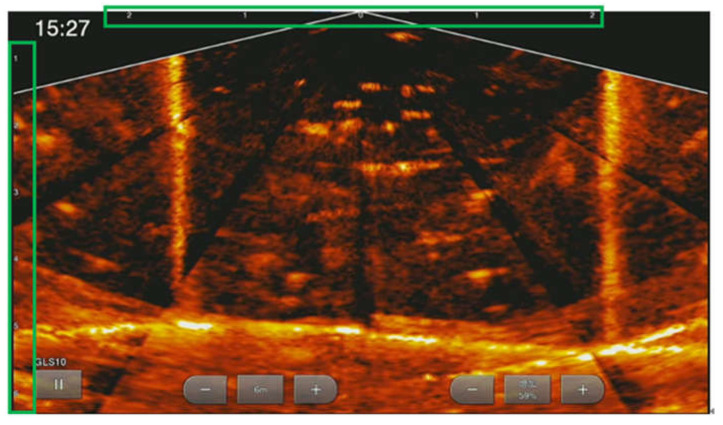
An example of the 1080×1092 sonar images captured from a 630 cm×600 cm fish pond.

Figure 9 shows an example of the 1080×1092 sonar images captured from a 630 cm×600 cm fish pond. Thus, each pixel in the sonar image would occupy an area of 0.583 cm×0.3125 cm in the fish pond. If a sonar pixel occupies $w\times h{\;\mathrm{cm}}^{2}$ in the fish pond, the 3D coordinates of the pixel $\vec{x}=\left(u,v\right)$ can be calculated as

(9) $$X_{S,\vec{x}}=\left[\begin{array}{ccc} x_{S}-\left(u*w\right), & y_{S}, & v*h \end{array}\right]$$

where $x_{S}$ and $y_{S}$ are the x-coordinate and the y-coordinate of the position of the sonar device in the world coordinate system. The value of $y_{S}$ equals to 0 when we set the origin of the world coordinate system to be [0, d, 0] where d is the depth of the sonar device. In practice, we can obtain the value of d by putting a depth sensor in the center of the sonar camera.

Given the pixel coordinates of A, B, C, D in the sonar image, Equation (9) thus generates the 3D point set $\left[X_{S,A},X_{S,B},X_{S,C},X_{S,D}\right]$. Similarly, the corresponding 3D point set $\left[X_{O,A},X_{O,B},X_{O,C},X_{O,D}\right]$ can be computed based on the four pixels in the stereo images using the disparity computing algorithm mentioned above. Equation (8) can now be written as

(10) $$\left[\begin{array}{c} \begin{array}{c} X_{O,A}\\ X_{O,B} \end{array}\\ X_{O,C}\\ X_{O,D} \end{array}\right]\;=R_{S\to O}\left[\begin{array}{c} \begin{array}{c} X_{S,A}\\ X_{S,B} \end{array}\\ X_{S,C}\\ X_{S,D} \end{array}\right]+T_{S\to O}$$

To solve the unknown parameters $\theta =(R_{S\to O},\;T_{S\to O}),$ we can apply any optimization scheme to minimize the following loss function:

(11) $$L_{\theta }=\frac{1}{4}\underset{i\in \left[A,B,C,D\right]}{\sum }{\left|\left|X_{O,i}-R_{S\to O}X_{S,i}+T_{S\to O}\right|\right|}_{2}$$

where ${\left|\left|X\right|\right|}_{2}$ is the L2 norm of the vector X. Although the object detection CNN for the common object detection has been proved to be accurate, the 2D coordinates of the detected matched points in both the sonar image and the stereo images still contain some errors. This error implies that the resulting 3D point pairs contain noises that reduce the reliability of the parameters θ. To further improve the quality of the learned parameters, the loss function for minimization can be rewritten as

(12) $$L_{\theta }=\frac{1}{4N}\sum_{i=1}^{N}\underset{j\in \left[A_{i},B_{i},C_{i},D_{i}\right]}{\sum }{\left|\left|X_{O,ij}-R_{S\to O}X_{S,ij}+T_{S\to O}\right|\right|}_{2}$$

where *N* is the number of frames used for parameter training.

### 3.3. Sonar and Stereo Camera Fusion for Fish Metrics Estimation

**Figure 10:**
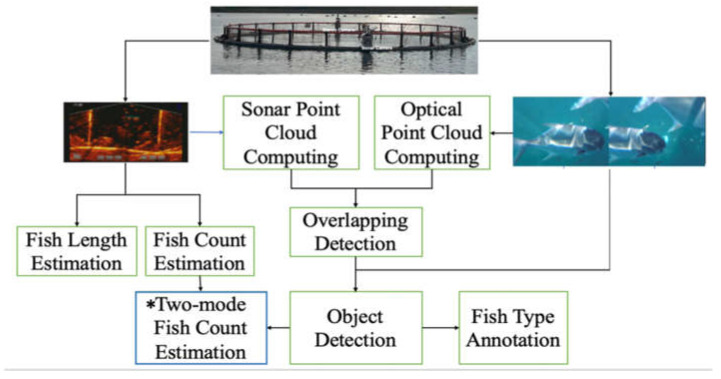
The fish metrics estimation uses sonar and stereo camera fusion and cloud-based AI functions.

**Figure 11:**
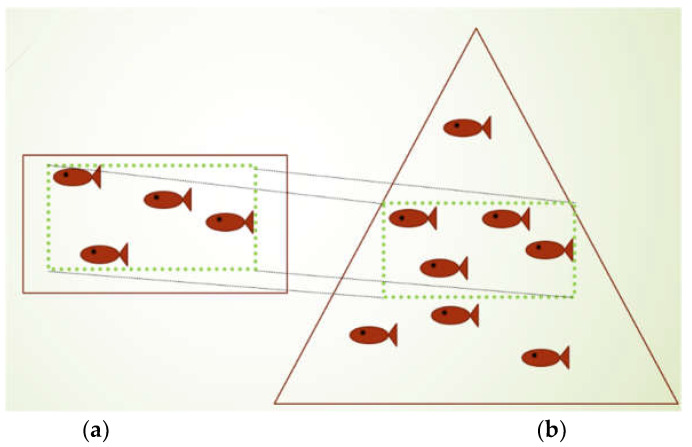
Schematic diagram of overlapping area detection.

Figure 10 shows the block diagram of the proposed fish metrics estimation using the sonar and stereo camera fusion and the cloud-based AI functions. First, as mentioned above, we can compute the 3D point clouds $P_{S}$ and $P_{O}$ for each captured sonar image $I_{S}$ and its synchronized stereo image pair $I_{O}$, respectively. Next, the overlapping detection module is applied to detect the area of the monitored fish pond or cage both cameras watch. Finally, the two 3D point clouds are inputted simultaneously into the overlapping detection module to identify their correspondence. Figure 11 shows the overlapping area of each camera system that was converted into the 3D point cloud discussed in the previous subsection. The two-mode fish count estimation could be an added feature to support the sonar camera for type-specific fish count estimation if the monitored fish pond or cage contains multiple types of fish.

The area covered by the optical camera is contained by the sonar device. Once the transformation parameters $\theta =(R_{S\to O},\;T_{S\to O})$ are obtained, the first step of our overlapping area detection is to compute the transformed point cloud:

(13) $${\dddot{P}}_{S}=R_{S\to O}P_{S}+T_{S\to O}$$

Next, we compute the overlapped point cloud:

(14) $${\hat{P}}_{S}=P_{S}\wedge {\dddot{P}}_{S}$$

For each 3D point ${\hat{X}}_{S,\vec{x}}=\left(x,y,z\right)$ in ${\hat{P}}_{S}$, we can then estimate the 2D coordinates of the pixel $\vec{x}=\left(u,v\right)$ as:

(15) $$\vec{x}=\left[(x-x_{S}\right)/w,z/h]\;$$

where *w* and *h* are the width and the height of a pixel in the sonar image, respectively; $x_{S}$ is the *x*-coordinate of the position of the sonar device in the world coordinate system. Finally, the bounding box $B_{S}$ to crop the sonar image is defined by the two corner pixels:

(16) $$\{\begin{array}{c} {\vec{x}}_{lu}=\left[\underset{{\vec{x}}_{i}\in {\hat{P}}_{S}}{\min}u_{i},\underset{{\vec{x}}_{i}\in {\hat{P}}_{S}}{\min}v_{i}\right]\\ {\vec{x}}_{rb}=\left[\underset{{\vec{x}}_{i}\in {\hat{P}}_{S}}{\max}u_{i},\underset{{\vec{x}}_{i}\in {\hat{P}}_{S}}{\max}v_{i}\right] \end{array}$$

#### 3.3.1. Estimation of Fish Standard Length and Weight Using Sonar Image

**Figure 12:**
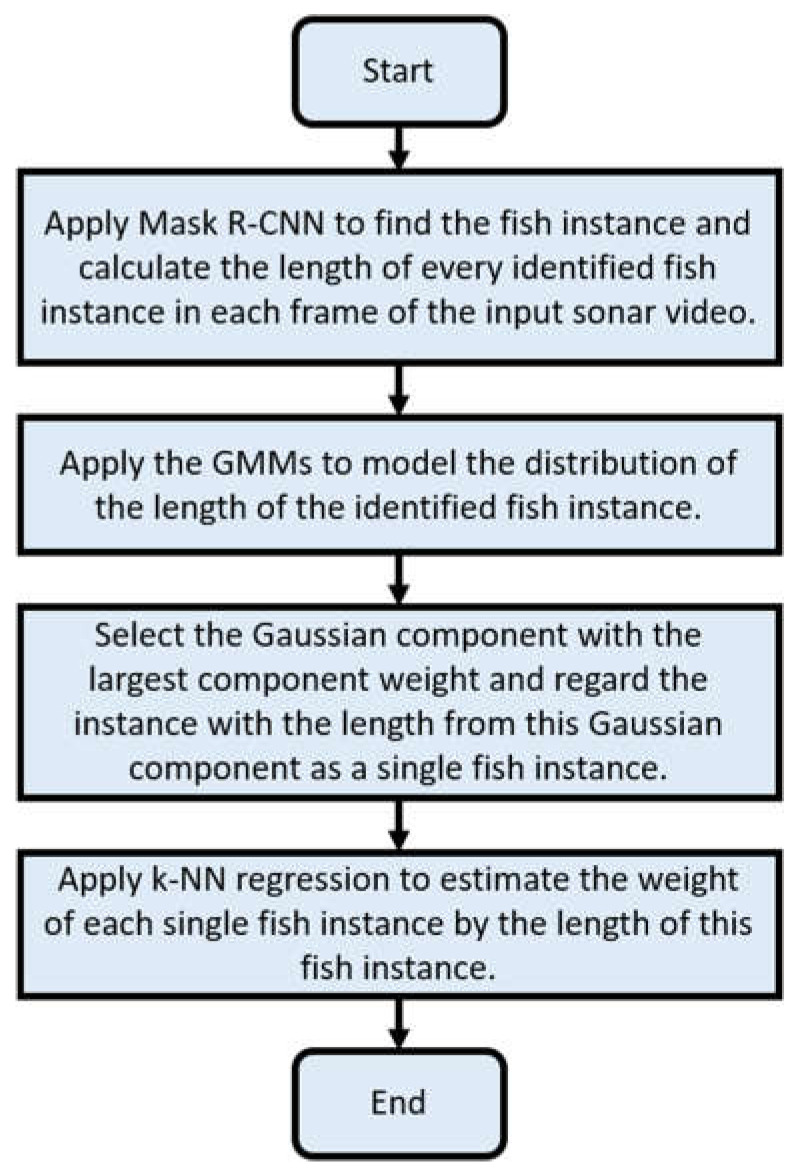
The flowchart to estimate the fish length and weight distribution.

The distributions of the standard length and weight of fish are essential to assessing the health and growth of the fish culture. As Figure 12 shows, there are four main steps for estimating those two distributions:

- Apply Mask R-CNN to identify fish instances in each frame of the input sonar video. The standard length of an identified fish instance is estimated by the distance between the two farthest points on this instance.
- Apply the EM algorithm [57] to learn a GMM for the distribution of the length of the identified fish instance. The GMM for the distribution of the length *x* can be expressed as follows:

(17) $$p\;\left(x\right)=\overset{c}{\underset{i=1}{\sum }}w_{i}\;N\;\left(x;\;\mu_{i},\;\sigma_{i}^{2}\right)$$

where c denotes the weight of the *i*th Gaussian components, $w_{i}$ denotes the weight of the *i*th Gaussian component, and $N\;\left(x;\;\mu_{i},\;\sigma_{i}^{2}\right)$ denotes the probability density of the Gaussian distribution with the mean of $\mu_{i}$ and variance $\;\sigma_{i}^{2}$. The probability of sample x from the *i*th Gaussian components, denoted by $p(G_{i}|x),$ can be estimated by:

(18) $$p(G_{i}|x)=\frac{w_{i}\;N\;\left(x;\;\mu_{i},\;\sigma_{i}^{2}\right)}{\sum_{i=1}^{c}w_{i}\;N\;\left(x;\;\mu_{i},\;\sigma_{i}^{2}\right)}$$

The sample x belongs to the *i*th Gaussian component $G_{i}$ if $p(G_{i}|x)$ is the largest among $p(G_{i}|x),\;i=1,\ldots ,c$. Given the number of Gaussian components c, the EM algorithm can find the parameters $w_{i,\;}\;\mu_{i}$ and $\sigma_{i}^{2}$ for each of the c components through maximum-likelihood estimation. In this paper, a non-Gaussianity criterion Φ (c) was defined in terms of the standardized skewness, and kurtosis [58] was adopted to determine the number c of Gaussian components:

(19) $$\Phi \;\left(c\right)=\frac{1}{c}\;\overset{c}{\underset{i=1}{\sum }}\left(\left|\frac{\frac{1}{\left|G_{i}\right|}\sum_{x\in G_{i}}{\left(x-\mu_{i}\right)}^{3}}{\sigma_{i}^{3}}\right|+\left|\frac{\frac{1}{\left|G_{i}\right|}\sum_{x\in G_{i}}{\left(x-\mu_{i}\right)}^{4}}{\sigma_{i}^{4}}-3\right|\;\right)\;$$

The EM algorithm was applied with the number of Gaussian components ranging from one to five. Then, the GMM with the least non-Gaussianity criterion Φ (c) was selected for the subsequent analysis.

- Select the Gaussian component $G_{i^{*}}$ with the largest component weight as the component comprising a single fish instance. Then, output the statistics of the fish length in $G_{i^{*}}$.
- Apply *K*-nearest neighbor regression with the training set, where the length and weight of the fish are measured manually to estimate the weight using the fish length in $G_{i^{*}}$. This paper set parameter *K* for the *K*-nearest neighbor regression to 5.

#### 3.3.2. Estimation of the Quantity of Fish in an Off-Shore Net Cage Using Sonar Image

The quantity $n_{fish}$ of fish in an off-shore net cage is estimated using the volume of the fish school that is swimming on the water surface and grabbing food pellets as follows:

(20) $$n_{fish}=\frac{V\times \delta }{V_{fish}}$$

where δ denotes the average fish density of the fish school, and V and $V_{fish}$ represent the volume of the fish school and the volume of the space occupied by a fish instance, respectively. In this paper, $V_{fish}$ is roughly estimated by $V_{fish}=l_{fish}\times \frac{l_{fish}}{2}\times \frac{l_{fish}}{2}\;$, which is the volume of the cuboid covering the space of a fish instance, where $l_{fish}$ denotes the average length of the fish instance and is measured beforehand. The volume V and the average length fish density d of the fish school are estimated by the average normalized pixel value of the fish region ℱ in the sonar image as follows:

(21) $$\delta =\frac{1\;}{g_{max}\times \left|ℱ\right|}\;\underset{x\;\in \;ℱ}{\sum }g\left(x\right)$$

where $g_{max}$ is the maximum pixel value in the fish region and |ℱ| denotes the number of pixels in ℱ.

**Figure 13:**
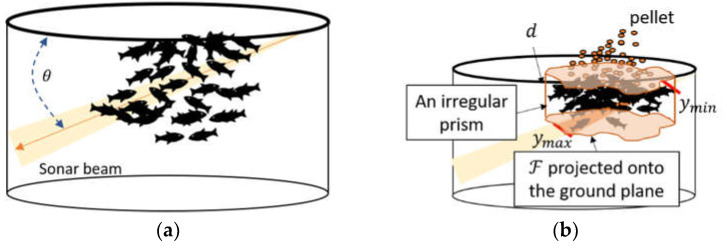
Illustration of the net cage and the fish school with the imaging sonar system, where (**a**) is the angle between the water and the plane of the sonar beam is θ; and (**b**) is the feeding fish school showing is grabbing pellets during feeding which is enclosed by an irregular prism.

There are several ways to scan the fish school to estimate their volume using the sonar system. For example, it can rotate and sideway scan the fish school. In this paper, for simplicity purposes, the fish school was analyzed without rotating the sonar beam. The sonar beam in Figure 13a passes through the fish school in a slantwise position, where the angle between the sonar beam and the seaplane is θ. Meanwhile, in Figure 13b, the space of the fish school when the fish is grabbing pellets is enclosed by an irregular prism, and the volume of the fish school is estimated by the volume of its irregular prism and can be expressed as:

(22) V=A×d

where A is the area of the fish regions in the sonar image projected onto the sea plane and d is the depth information of the fish school.

**Figure 14:**
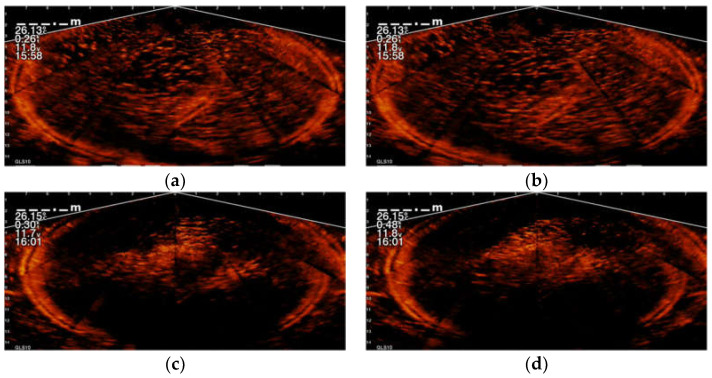
Sonar images of a fish school in an off-shore net cage, where (**a**) and (**b**) show the fish dispersing; and (**c**) and (**d**) show fish swimming toward the water surface to grab feed pellets.

The pattern of the fish school in the sonar image when the fish gathers and swims toward the fish surface to grab the pellets is different compared with when the fish disperses, as shown in Figure 14. In this work, a CNN is first applied to find the frame in the sonar video where the fish school gathers and grabs the pellets. Then, the fish region ℱ in the said frame is identified using a semantic segmentation network; the details of these two neural networks will be presented later. The area A of the bottom of the prism and the depth of the fish school can be estimated by:

(23) $$A=\;|ℱ|\times \;{∆}_{x}\times {∆}_{y}\times \cos\left(\theta \right),\;d=\;y_{max}\times \;{∆}_{y}\times \sin\left(\theta \right)$$

where $\;y_{max}$ denotes the bottom row of this region and $\;{∆}_{x}$ and $\;{∆}_{y}$ denote the width and height of a pixel in centimeters, respectively.

**Figure 15:**
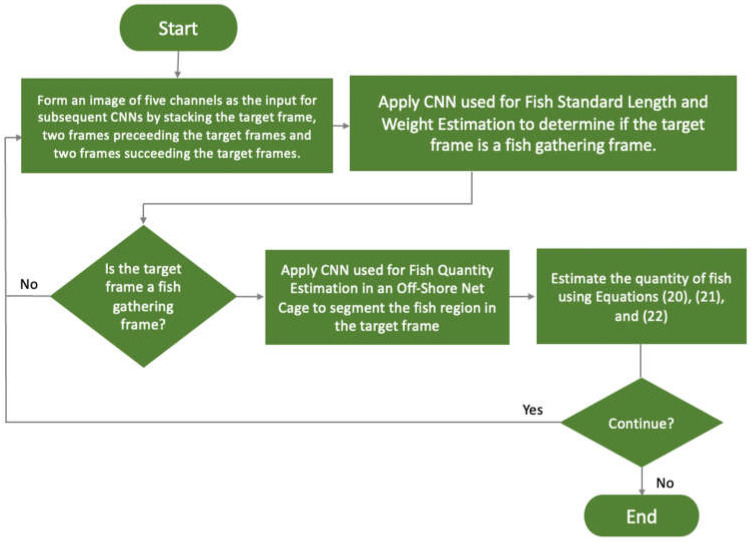
The flowchart of estimating the quantity of fish in an off-shore net cage.

Figure 15 shows how to estimate the quantity of fish in an off-shore net cage. For the first step, it constructs the input for the subsequent two CNNs by stacking five successive frames to form a five-channel image. These five frames are the target frame, two preceding, and two succeeding frames of the target frame. Next, the CNN presented in Section 3.3.3 is applied to determine if the given frame is a fish-gathering frame. If the target frame is classified as a fish-gathering frame, the CNN in Section 3.3.4 is applied to segment the fish region in the target frame. Equations (20), (21), and (22) are then used to estimate the fish quantity. The neural network architectures of the two CNNs are described in the succeeding subsections.

#### 3.3.3. CNN for Detecting the Fish-Gathering Frame

**Figure 16:**
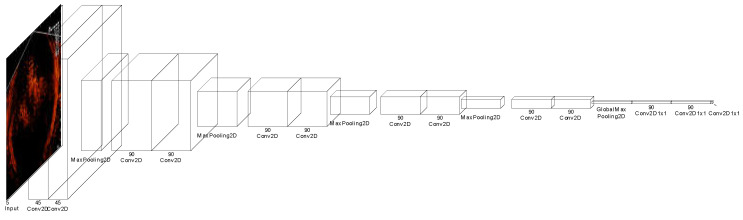
The neural network architecture for detecting fish-gathering frame.

Figure 16 shows the neural network architecture of the CNN for detecting the fish-gathering frames. The input for the CNN is a five-channel image comprising five successful sonar image frames. The kernel size of the first ten convolutional layers of the CNN is all of size 3 × 3 with a corresponding activation function ReLu. Meanwhile, the last three layers also have a ReLu activation function, and a sigmoid was incorporated into the last 1 × 1 convolution layer.

#### 3.3.4. Semantic Segmentation Network for Segmenting Fish Regions

**Figure 17:**
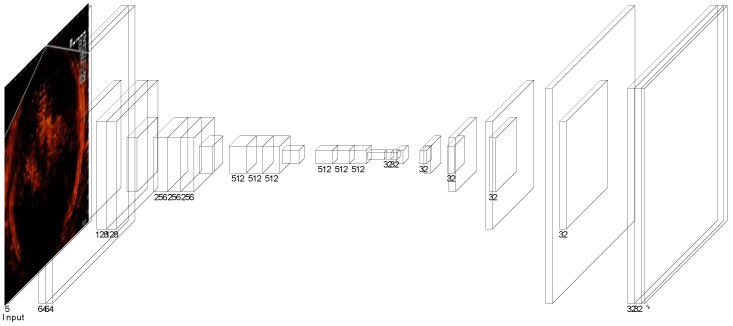
The neural network architecture for segmenting fish regions in the sonar image.

The semantic segmentation networks’ neural network architecture to segment fish regions in the sonar image is shown in Figure 17. The neural network is based on the U-Net architecture [59]. The input of this CNN is a five-channel image comprising five successive sonar image frames. In the semantic segmentation network, the transposed convolutional layer with strides (2,2) is adopted for up-sampling. The kernel size of the convolutional layer and the transposed convolutional layer is 3 × 3. The activation of the last later is softmax, and the activation function of the other layers ReLu.

### 3.4. Object Detection for Fish Type Identification and Two-Mode Fish Counting

The fish of the left image of the input stereo image pair is detected by any object detection CNN, e.g., the YOLOv4. The object detection results can be used to annotate the fish type since the types of fish are given in the training dataset to train the object detection CNN. Let $c_{i}$ and $c_{total}$ be the fish count of the *i*-th type and the total count of fish detected based on the RGB image. As mentioned above, the sonar image estimates the number of fish without information on fish types. To deal with the difficulty, our two-mode fish counting algorithm estimates the count of the *i*-th type fish as:

(24) $$C_{i}=C_{sonar}\times c_{i}/c_{total}$$

In this study, we focused on the design of the two-mode smart sensor, which consists of a sonar scanning device and a stereo optical camera. The captured images are sent to the cloud using a wireless communication network. Although the object detection CNN is not new, we can design a new CNN architecture for underwater object detection to improve the accuracy of fish distributions using (24). Note that the functionality of the smart sensor is incremental since we can add a new AI function into the cloud to provide a new service for sensor fusion.

## 4. Experimental Results

### 4.1. Sonar and Stereo Camera Fusion Results

**Figure 18:**
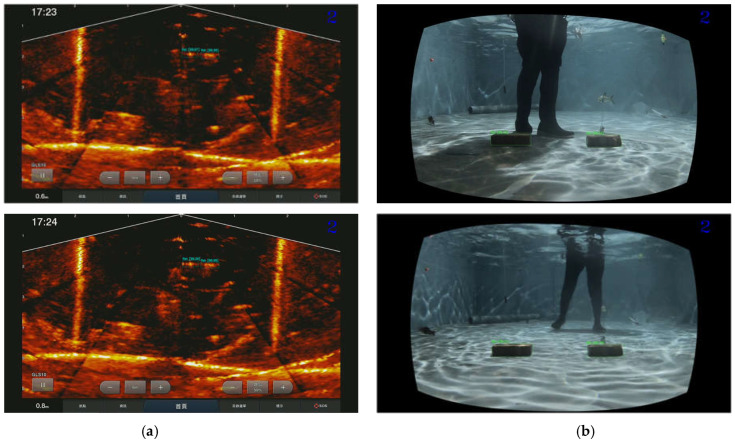
Detection results (marker) of the target object from (**a**) sonar and (**b**) stereo images.

The fish objects detected in the sonar images are mapped into the stereo camera image as an area of interest. In Figure 18, we conducted an experiment using the two bricks as the target object representing the fish to determine the object detection capability of our proposed method. Of course, sonar images have a wider range than stereo images, and the positions of the target objects are entirely different. However, based on the detection results for both camera systems, our approach identified the target objects using different sonar and stereo image frames.

**Figure 19:**
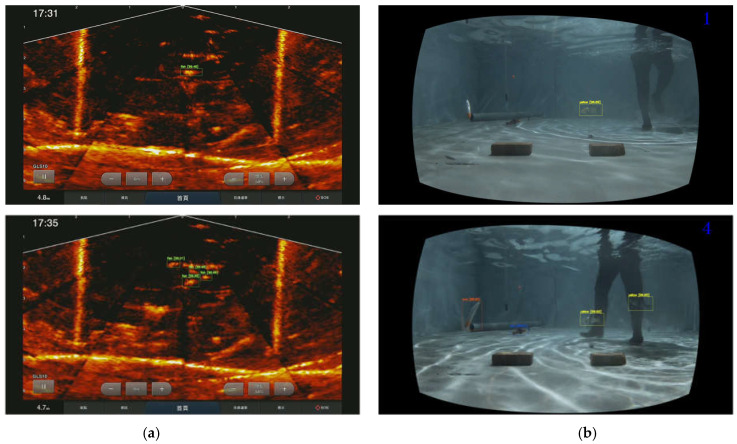
Fish classification results using (**a**) sonar and (**b**) stereo images.

On the other hand, the fish detection in Figure 19a identified the same number of fish objects in the sonar image and are all correctly mapped and detected with fish annotated in the stereo images in Figure 19b.

**Figure 20:**
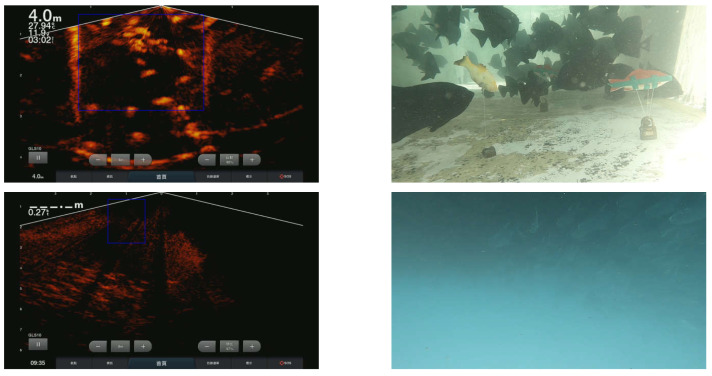

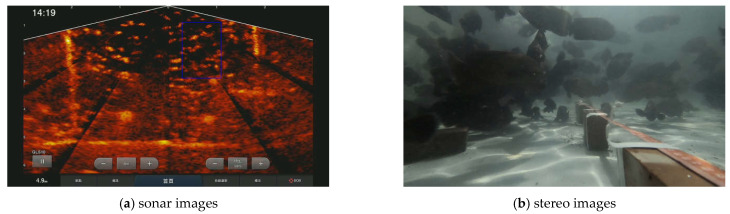
Bounding box detection results from the sensor fusion.

To ensure that our mechanism detects a correct object in both sonar and stereo images, we integrated a bounding box that shows the area covered by the stereo image in the sonar image in Figure 20, where a shows the sonar image with its corresponding area covered in the stereo image while b shows the entire stereo image area that appears in some part of the sonar images. The images were taken from our various aquaculture locations.

### 4.2. Estimation of Fish Standard Length and Weight Using Sonar Images

**Figure 21:**
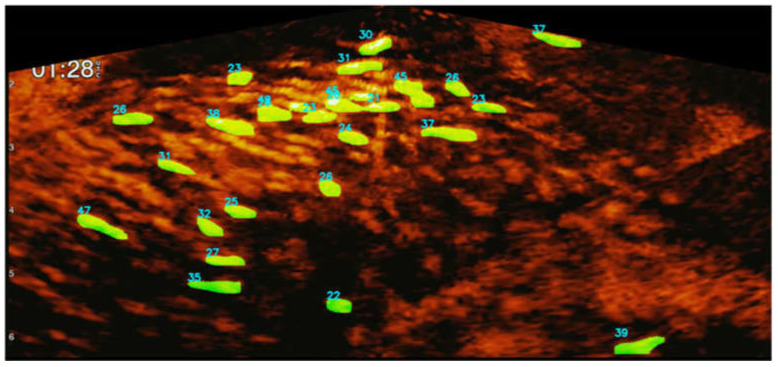
Fish detection using Mask R-CNN and length estimation results using the sonar camera system.

Figure 21 shows the fish instances of a sonar image detected by Mask R-CNN. Table 1 shows that the true positive rates of Mask R-CNN for the three experimental environments were approximately 85, 90, and 75%, respectively. Environment C incurred the lowest positive rate, which was affected by the crowded environment of the fish cage. The number values in the image represent the fish length estimation.

**Figure 22:**
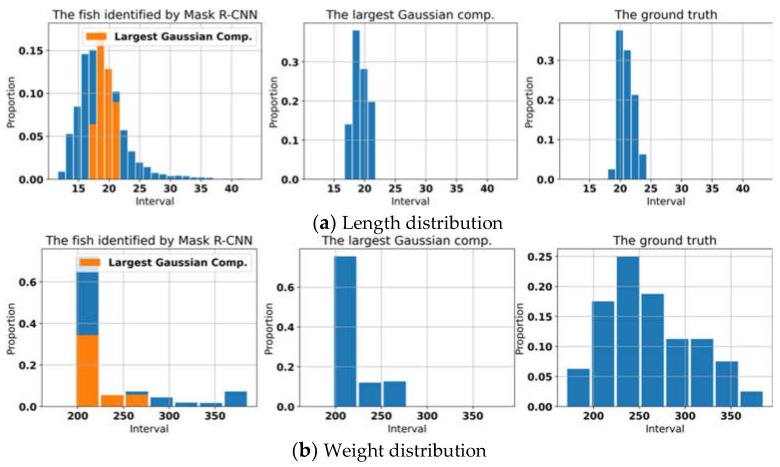
The distribution of fish length and weight, identified by Mask R-CNN, largest Gaussian component, and the distribution for data manually measured or ground truth.

Table 2, on the other hand, shows the experimental results where the relative errors of the average length and weight can be reduced by applying GMMs. The length and weight of each fish in the tank were measured in all environments. We compared the distributions of all estimated data, the estimated data incorporating GMM, and the ground truth (Figure 22), all presented in Table 2. The t-test and Bartlett test were used to determine if the distributions of the two independent samples were significantly different or not in terms of means and variances, respectively. The comparison result showed that the length distributions of the three data were different in means. The p-values for the ground truth vs. GMM, and the ground truth vs. the distribution of fish length identified by Mask R-CNN were $1.11\times 10^{-41}$ and $2.57\times 10^{-8}$, respectively. However, the variance of the fish length processed by the GMM and the ground truth was similar (the *p*-value is 0.61 and the p-value for the other pair is $1.12\times 10^{-24}$). In manually measuring the fish length, we used the fork length.

### 4.3. Estimation of Fish Quantity in a Net Cage Using Sonar Images

**Figure 23:**
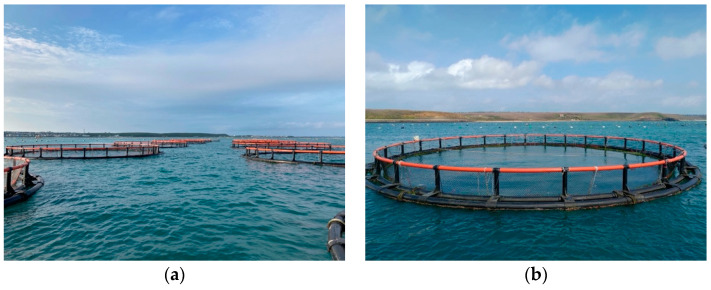
Off-shore cages in Penghu, Taiwan, where (**a**) shows the landscape of the off-shore net cages; and (**b**) is the environment of the net cage used for the experiment.

Figure 23 shows the off-shore net cage environment in Penghu, Taiwan, for the fish quantity estimation with Trachinotous blochii as the fish species. The net cage is 15 m in diameter and 5 m in depth, with approximately 2200 fish instances during the experiment. The average standard length of the fish was 20 cm, and the sonar beam was positioned at a slant angle θ of 20°.

The dataset used to train the CNN in detecting fish-gathering frames consisted of 58 fish-gathering images and 116 fish-dispersing images. The CNN was evaluated using 10-fold cross-validation and obtained an accuracy of 0.98. Table 3 shows the confusion matrix results. The intersection over union (IoU) was adopted to represent the performance index for the semantic segmentation network to segment the fish region. This network was evaluated using 10-fold cross-validation, and the average IoU was 0.77 ± 0.66.

**Figure 24:**
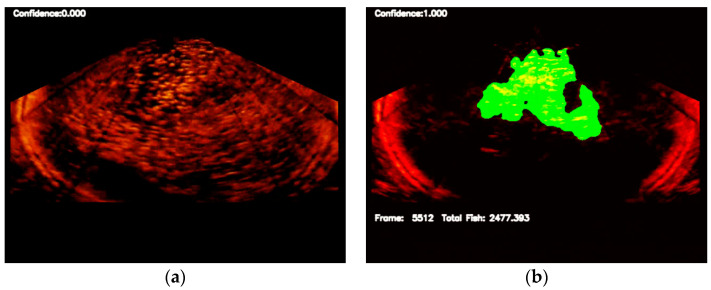

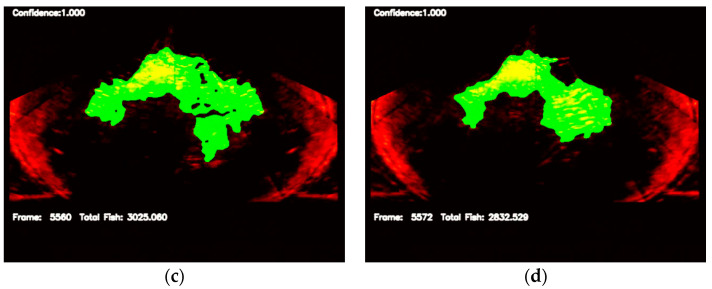
Experimental results of estimating fish quantity where (**a**) shows the non-gathering characteristics of the fish school; and (**b**–**d**) show the gathering feature of the fish school and the estimated fish quantity based on the fish region identified by the semantic segmentation network.

**Figure 25:**
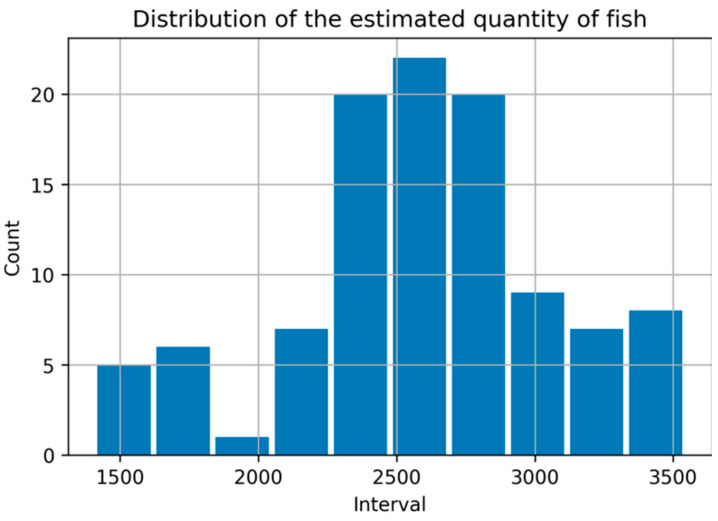
The distribution of the estimated fish quantity.

Figure 24 shows the results of applying the procedures in Figure 15 for fish quantity estimation. The quantity of the fish was estimated using 105 fish gathering frames. Figure 25 shows the distribution of the estimated fish quantity with a mean and standard deviation of 2578.72 and 569.099, respectively. Therefore, the manual estimation of the quantity of fish in the net cage was 2200, within the estimate’s 68% and 95% confidence intervals with values of [2112.32, 3045.21] and [1659.33, 3498.11], respectively.

### 4.4. Object Detection for Fish Type Identification and Two-Mode Fish Count

**Figure 26:**
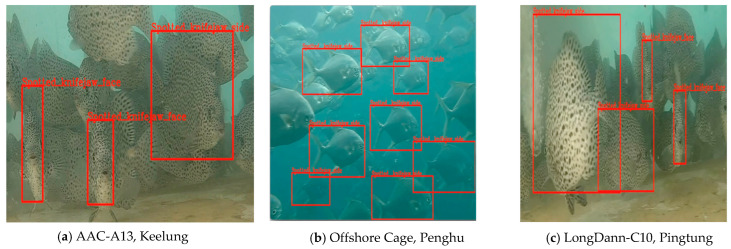
Fish target object detection results using YOLOv4 in the different aquaculture sites.

**Figure 27:**
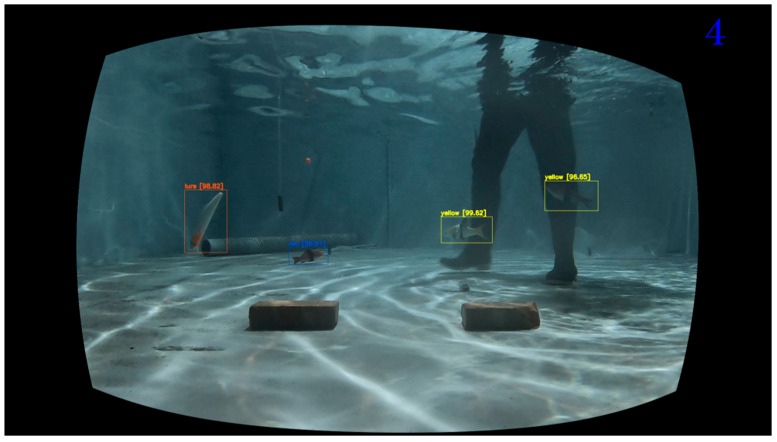
Fish target object detection results using YOLOv4 using the fake fish experiment.

The object detection model utilized the pre-trained YOLOv4 with the COCO dataset [60] was used for the object detection model. The object detection results representing three different aquaculture environments (Keelung, Penghu, and Pingtung locations) are shown in Figure 26. The experimental result for the fake fish experiment is in Figure 27, where three fish species were detected.

**Figure 28:**
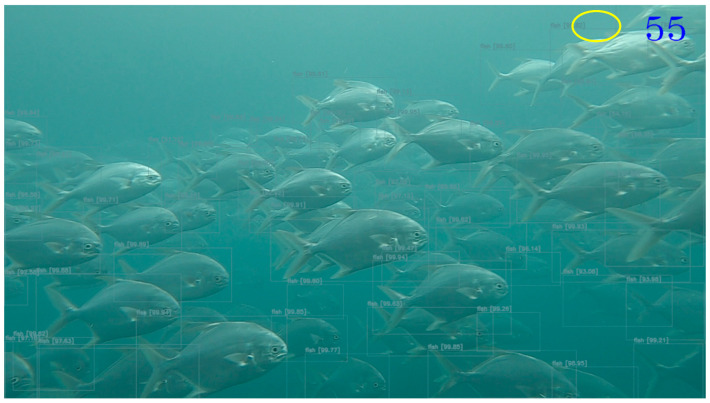
Stereo camera system trachinotus blochii species count estimation.

Figure 28 is the result of the fish count estimation, which shows the actual Trachinotus blochii species detected from the images taken from the Penghu off-shore cage. Since the low-cost stereo camera range is short, it cannot see other fish objects beyond its reliable area coverage; thus, only 55 fish objects were detected and counted.

## 5. Discussion

Our sensor-based fusion mechanism was applied to aquaculture monitoring using optical (stereo camera) and sonar images. Each sensor system has its strength and limitations, and we took advantage of its capabilities to address the issues of the other sensors. For example, it would be difficult for an RGB camera to accurately estimate fish metrics due to poor underwater conditions addressed by the sonar camera system. Therefore, we took advantage of the texture information of the optical images to provide fish species annotation to sonar images. In addition, detecting the common area of each sensor poses a significant challenge considering the quality of images in the underwater environment.

Additionally, sonar cameras have a larger area covered when compared with stereo cameras. Thus, the target objects will be in different positions or locations. We also must consider that sensors vary in terms of errors, the origin of the coordinate axis, and the types of data received. Two essential issues need to be addressed for sensor fusion, namely, opti-acoustic extrinsic calibration and opto-acoustic feature matching [14]. To deal with this and improve the quality of our data fusion, we performed camera calibration to enable both sensors to be in a common world frame or coordinates. Our approach integrated the 3D point cloud information of both sensors to identify the overlapping areas by using markers (4 pixels) to project sonar images to stereo images as part of the learning phase. The integration of the transformation matrix made it possible to locate the corresponding pixels in both camera systems. Camera calibration performed a significant function in our sensor fusion by transforming their corresponding rotation matrix and translation vectors to match the features from sonar to optical coordinate system, thus taking advantage of the epipolar geometry for the multi-modal feature association [14]. For opti-acoustic feature matching, the 3D information was utilized to identify the same features of both sensor modalities.

One of the AI functions is to estimate the length and weight distribution of the fish in an indoor aquaculture tank. The main challenge of the first method is that the fish in the aquaculture tank are often crowded and overlapped in sonar images. Besides, sonar images of an aquaculture tank are usually noisy due to the echo from the air pumper and the bottom and wall of the tank. The Mask R-CNN [61] identified single fish instances in sonar images. Therefore, it may locate overlapped and incomplete fish for crowded aquaculture tanks as single fish instances. Because Mask R-CNN determines the fish instance, which looks like a single fish instance, an assumption that the length distribution of identified instances is a mixture of Gaussians and the length distribution of the valid single fish instance is the largest Gaussian component of this mixture of Gaussians. Based on that assumption, the first method employs Gaussian mixture models (GMMs) to model the length distribution of the fish instance identified by Mask R-CNN. Then, the proposed method regards the fish instance with the length from the Gaussian component with the largest mixture component weight as a single fish instance and estimates the weight of the instance by the *k*-nearest-neighbor regression. Since we manually measured the fish fork length as the basis for the length estimation, our proposed estimation method is biased since the fork length is usually more significant than the standard length.

Furthermore, the response of the caudal fish fin in the sonar image is usually weak, which makes the fish length measured by our proposed method close to the standard length of the fish. On the other hand, the estimated weight distribution and the ground truth were significantly different. This result could be attributed to the fish’s weight being affected by other factors, such as the thickness of the fish. In practice, it is difficult to observe both the thickness and length of the fish instance from the view of the imaging sonar system. Overall, the relative error of the estimated average fish standard length was approximately 15%, while the relative error for the estimated average weight was less than 50%.

The second AI function for sonar images estimates fish quantity in an off-shore net cage, and we identified two main challenges. First, the fish considered in this paper, which were Trachinotus blochii, are often widely distributed in the net cage. Second, the view of an imaging sonar system only covers a small portion of the net cage. Since the fish of the target species can gather and swim close to the water surface to grab food pellets, the proposed method only estimates the quantity of fish in the net cage when feeding fish. The number of fish is assessed on the average fish volume and the fish school’s estimated volume and density. In this paper, a convolutional neural network was developed to determine if fishes gathered and were grabbing food pellets. Then, we supposed that the fishes gathered and were grabbing feed. In that case, a semantic segmentation network was applied to segment the fish school in the sonar image, and the volume of the fish school was estimated on the segmentation result. The visible imaging has a short imaging distance underwater due to the light attenuation caused by water absorption and scattering. Therefore, the image was more blurred, and the quantity of the image decreased as the shooting distance increased. However, the sound wave can travel far through water without attenuation. Consequently, counting based on acoustics can still work when visual counting is inappropriate [62].

For object detection, this work used two images from the two sensors to capture a common target. The YOLOv4 [55], with an efficient and powerful object detection model, which makes it possible to achieve real-time object detection, was used to identify or detect the target object in the sonar and stereo images. In the two-mode fish counting estimation, we detected the type of fish found in the cage and provided an assessment of the number of populations for each species. Since we used a low-cost camera type, its range is minimal, so it cannot detect fish out of its range even with robust object detection deep learning models such as YOLOv4. Therefore, we only counted detected fish images within the reliable range, which is why we had a lower fish count when compared with the estimated number of fish in the cage, unlike the sonar camera system that covers a larger and broader range. Thus, we will still rely on the sonar camera system for the final fish population count to perform such estimation. The two-mode fish counting estimation now serves as a sampling device to support and assist the sonar camera system in providing information about the fish species distribution as added sonar image analytics. However, at the moment, the dataset available is only one species per cage/pond. However, we tested our mechanism to perform annotation functions using a well-known object detection CNN to check if our proposed method can detect various fish types. We can replace the YOLOv4 with a state-of-the-art object detection CNN because YOLOv4 does not work well in the underwater environment. This shortcoming will be one of our future works to improve the performance of our smart sensor fusion.

The success of the data collection procedure for this study poses a difficult task. First, the assistance of the aquaculture operators is much needed during the data collection, and they must be present in every data collection activity. Second, the data can only be obtained when the aquaculture operators feed the fish, usually once a day. Third, the weather is another major factor since the net cages are in the open sea. Finally, it is essential to consider the sea current to make sure that the transducer of the imaging sonar system is steady since it greatly affects the quality of the data. After several attempts to collect data, only a few were collected due to difficulties.

## 6. Conclusions

The 3D point clouds for each camera system were separately obtained, extracted, and matched to find each correspondence. Our sensor fusion approach detected the corresponding pixels or the bounding box of the common area. Thus, it could also detect the fish objects in the images of both sensors to be utilized for fish type annotation. In this paper, two methods were developed to estimate the quantity and the distribution of the standard length and weight of fish using a sonar imaging system. The first method was developed for estimating the distribution of the standard length and weight of fish. Using GMMs to find the distribution of the standard length of single fish instances and employing K-nearest-neighbor regression to estimate the weight of fish by the length, the relative errors of the estimated average fish standard length and weight were approximately less than 15 and 50%, respectively. Those errors can be reduced if the fish length manually measured is based on the standard length instead of the fork length. Therefore, the proposed method can be applied to monitoring the growth of culture fish. The second method estimates the fish quantity in an off-shore net cage. The preliminary experimental result showed that the quantity of fish could be within the estimate’s 68 and 95% confidence intervals. The 68 and 95% confidence interval widths were approximately 900 and 1800, respectively. Preliminary experimental results showed that the proposed method is feasible. Lastly, the fish target object detection provides an additional function to annotate fish species and offers additional information to the sonar system. For our future works, we plan to incorporate Generative Adversarial Networks to convert the optical image of the target object into a sonar image. Additionally, we will integrate sonar and stereo camera fusion for fish length and weight estimation.

## Figures and Tables

**Table 1:** The true positive rate of Mask R-CNN for different experimental environments.

Environment	True Positive Rates
A	85%
B	90%
C	75%

**Table 2:** The relative error of the estimated average standard length and weight of fish, where the ground truth for the average standard length and weight of fish is measured manually, and *ε*, *N*, *NG*, and *c* denotes the relative error, the number of fish instances identified by Mask R-CNN, the number of the instance in the largest Gaussian component, and the number of Gaussian components, respectively.

Environment	Length (cm)					Weight (g)					*N*	*N_G_*	*c*
	Manual	w/o GMM		w/GMM		Manual	w/o GMM		w/GMM				
		ℓ	ε	ℓ	ε		w	ε	w	ε			
A (month A)	21.98	8.82	0.14	19.29	0.12	286.37	236.45	0.17	220.32	0.23	4, 456	2, 020	4
A (month B)	21.98 ^†^	27.13	0.24	23.27	0.06	286.37 ^†^	337.39	0.18	315.49	0.10	1, 911	1, 319	2
A (month C)	21.98 ^†^	23.77	0.08	22.31	0.01	286.37 ^†^	315.44	0.10	297.75	0.04	210	168	2
A (month D)	21.98 ^†^	25.16	0.14	22.63	0.03	286.37 ^†^	335.13	0.17	305.94	0.07	33	21	2
A (month E)	21.98 ^†^	24.38	0.11	22.21	0.01	286.37 ^†^	316.57	0.11	296.78	0.04	2, 413	1, 879	2
B (month A)	18.01 ^†^	20.18	0.12	18.90	0.05	180.12 ^†^	210.93	0.17	201.03	0.12	1, 201	1, 034	2
B (month B)	18.01 ^†^	20.30	0.13	19.26	0.07	180.12 ^†^	218.01	0.21	210.78	0.17	3, 279	1, 448	5
B (month C)	18.01 ^†^	21.16	0.18	21.16	0.18	180.12 ^†^	228.60	0.27	228.60	0.27	1, 1228	1, 228	1
C (month A)	15.46	19.78	0.28	17.98	0.16	171.66	272.13	0.59	249.82	0.46	12, 101	9, 781	2
C (month B)	15.50	19.79	0.28	17.51	0.13	173.90	262.86	0.51	236.27	0.36	14, 488	11, 165	2
C (month C)	16.13	16.13	0.22	17.58	0.09	189.36	262.84	0.39	235.98	0.25	10, 121	7, 814	2
C (month D)	16.33	16.33	0.20	18.03	0.10	211.43	290.57	0.37	269.56	0.27	8, 480	7, 013	2

Note. ^†^: no measurement of data available; data was based on the previous months.

**Table 3:** The confusion matrix for the CNNs’ detection of fish gathering frame.

Actual	Predicted	
	Gathering	Dispersing
Gathering	58	0
Dispersing	3	113

## Data Availability

Not applicable.

## Abbreviations

AIoT	Artificial Intelligence-based Internet of Things
CNNs	Convolutional Neural Networks
EM	Expectation Maximization
GMM	Gaussian Mixture Model
IoT	Internet of Things
K-NN	K-Nearest Neighbors
ReLU	Rectified Linear Unit
RGB	Red, green, blue
YOLOv4	You only look once version 4
